# Antiviral Gene Expression in Young and Aged Murine Lung during H1N1 and H3N2

**DOI:** 10.3390/ijms222212097

**Published:** 2021-11-09

**Authors:** Rebecca Harris, Jianjun Yang, Kassandra Pagan, Soo Jung Cho, Heather Stout-Delgado

**Affiliations:** Weill Cornell Medicine, Division of Pulmonary and Critical Care, New York, NY 10021, USA; rebecca.m.harris@cuanshutz.edu (R.H.); jiy2017@med.cornell.edu (J.Y.); klp3002@med.cornell.edu (K.P.); sjc9006@med.cornell.edu (S.J.C.)

**Keywords:** antiviral, H3N2, H1N1, influenza, aging, lung

## Abstract

Influenza is a respiratory virus that alone or in combination with secondary bacterial pathogens can contribute to the development of acute pneumonia in persons >65 years of age. Host innate immune antiviral signaling early in response to influenza is essential to inhibit early viral replication and guide the initiation of adaptive immune responses. Using young adult (3 months) and aged adult mice infected with mouse adapted H1N1 or H3N2, the results of our study illustrate dysregulated and/or diminished activation of key signaling pathways in aged lung contribute to increased lung inflammation and morbidity. Specifically, within the first seven days of infection, there were significant changes in genes associated with TLR and RIG-I signaling detected in aged murine lung in response to H1N1 or H3N2. Taken together, the results of our study expand our current understanding of age-associated changes in antiviral signaling in the lung.

## 1. Introduction

Influenza A virus (IAV) is a respiratory virus that alone or in combination with secondary bacterial pathogens can contribute to the development of acute pneumonia in persons >65 years of age [1,2]. Historically, influenza has been recognized as one of the leading causes of respiratory tract infection and contributes to seasonal epidemics. Due to antigenic variation and interspecies transmission, influenza remains a threat to global health.

Production of type I interferons (IFN) represents one of the key lines of defense against influenza. Host innate immune antiviral signaling early in response to influenza is essential to inhibit early viral replication and guide the initiation of the adaptive immune response. Infection with influenza results in the production of pathogen-associated molecular patterns (PAMPs), which are recognized by pattern recognition receptors (PRR). Several PRRs, such as Toll like receptors (TLR), NOD-like receptors (NLR), and retinoic acid inducible gene-I (RIG-I) like receptors (RLR) play a key role in the activation of the antiviral immune response to influenza. TLR 3 and 7 recognize double stranded (dsRNA) and single stranded (ssRNA) RNA, respectively and can initiate TIR domain-containing adaptor inducing IFN-β (TRIF) or myeloid differentiation factor-88 (MyD88) dependent antiviral signaling [3,4,5,6,7,8]. In addition, RLR, which recognize invasive viral RNA produced during infection and, through induction of mitochondrial antiviral signaling (MAVS) molecule, can also induce type I IFN production [9,10,11]. Expression of additional signaling molecules, such as TRIM25 which induces MAVS complex formation with DExD/H-box helicase (DHX33), or MAVS mediated recruitment of TNF-receptor-associated factor 3 (TRAF3) and subsequent activation through TANK-binding kinase-1 (TBK1), can further amplify these signaling cascades in response to influenza [10,11,12]. Upregulation of type I IFN by multiple lung cell types, such as macrophages, dendritic cells (DC), plasmacytoid DC, and epithelial cells, and subsequent binding to IFN-α/β receptors (IFNAR) present on the surface of infected neighboring cells can further stimulate antiviral responses [13]. Interaction of type I IFNs with their cognate receptors contributes to the recruitment and activation of additional signaling molecules, such as signal transducer and activator of transcription (STAT) 1 and 2 [14,15]. 

Previous work has demonstrated a decline in TLR function and cellular immunity in aging that contributes to increased susceptibility of older persons to influenza [16,17,18,19,20,21,22,23,24,25,26]. Similarly, defects in RIG-I signaling, which contribute to cell-intrinsic defects in IFN induction, have been observed in older adults [27]. While various age-associated defects have been associated with immunosenescence and impaired CD4 and CD8 T cell mediated responses, increased basal levels of various inflammatory cytokines and chemokines also contribute to diminished and/or dysregulated immune capacity in the aged lung [16,26,28,29,30,31,32,33,34]. In addition to reduced T cell activation, experimental studies have illustrated age-associated defects in several key components of the immune response, such as reduced antigen-capture capacity, impaired migration of DCs, decreased IFN-γ production by natural killer (NK) cells, and reduced cytokine production [16,17,28,35,36,37,38,39,40]. The goal of our current work is to provide a greater insight of antiviral signaling at days 3, 5, and 7 of H1N1 or H3N2 infection in young and aged adult murine lung. Using a murine model of mouse adapted H1N1 and H3N2 infection, the results of our study illustrate dysregulated and/or diminished activation of key signaling pathways in aged lung contribute to increased lung inflammation and morbidity. Taken together, the findings presented expand our current understanding of age-associated changes in antiviral signaling in the lung.

## 2. Results

As innate antiviral signaling in the lung plays a pivotal role in mediating host innate immune responses to influenza, the purpose of our current study was to examine the impact of chronological aging on antiviral signaling in young and aged adult lung in response to mouse-adapted strains of H1N1 and H3N2.

### 2.1. Impact of Chronological Aging on Host Responses to Mouse-Adapted H1N1 and H3N2 Strains of Influenza

#### 2.1.1. Morbidity and Histological Changes in the Young and Aged Murine Adult Lung 

**Figure 1:**
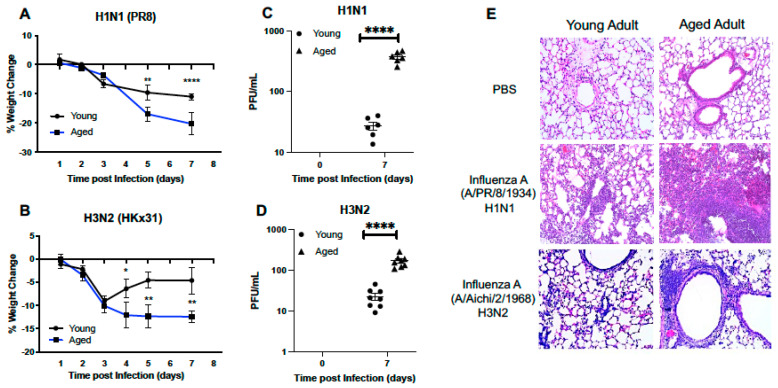
Morbidity and Histological Changes in the Young and Aged Murine Adult Lung. Young (3 months) and aged (18–20 months) adult mice were intranasally instilled with 12.5 PFU of influenza (strain: A/Puerto Rico/8/1934, PR8, H1N1) or (strain: A/Aichi/2/1968, HKx31, H3N2). Weight measurements were taken for young and aged mice at select time points post (**A**) H1N1 or (**B**) H3N2 infection. Viral titer in BAL was quantified and PFU/mL for (**C**) H1N1 and (**D**) H3N2. (**E**) Lung tissue was collected at day 7 post infection and H&E staining was performed to assess inflammation and cellular recruitment to lung. For H1N1 and H3N2 samples, each tissue section (20X magnification) shown represents a different mouse. Student’s *t*-test: * *p* < 0.05, ** *p* < 0.01, and **** *p* < 0.0001. Similar results were obtained from at least three independent experiments, with N = 10 per group. Data are expressed as the mean ± SD.

To understand if there were age-associated differences in the host response to influenza, we infected young (3 months) or aged-adult (18–20 months) mice with mouse-adapted strains of influenza (strain: A/Puerto Rico/8/1934, PR8, H1N1) or (strain: A/Aichi/2/1968, HKx31, H3N2). In response to both strains of influenza, significantly sustained loss of weight was observed in aged adult mice when compared to young (Figure 1A,B). When compared to young, there was also a significant increase in viral titer present in aged lung in response to either H1N1 (Figure 1C) or H3N2 (Figure 1D). Histological examination of lung tissue illustrated increased cellular infiltration, with a notable increase in inflammation and intra-alveolar edema detected in aged adult lung at day 7 of infection (Figure 1E). In response to H1N1, there was increased cellular recruitment to aged adult murine lung, with detectable damage to the alveolar capillary barrier (Figure 1E). Interestingly, when compared to lung tissue collected from aged H1N1 infected mice, there was less inflammation and inflammatory damage in aged H3N2 infected lung detected at day 7 post infection (Figure 1E).

#### 2.1.2. Cellular Infiltration and Lung Injury Is Increased in Aged Murine Lung in Response to Influenza

**Figure 2:**
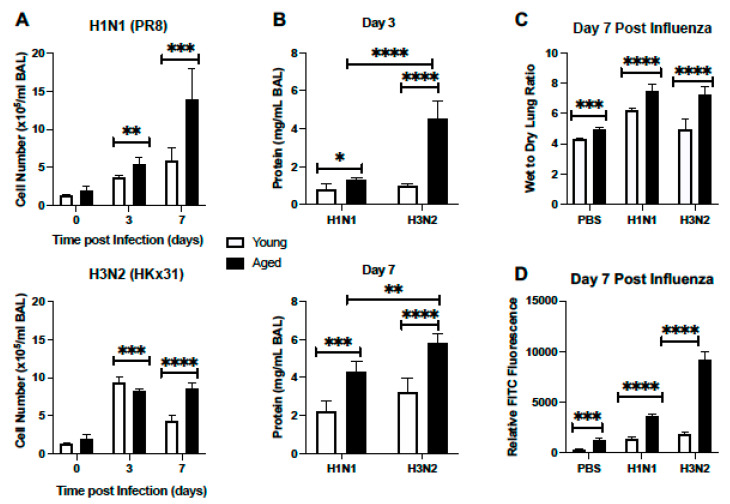
Cellular Infiltration and Lung Injury is Increased in Aged Murine Lung in Response to Influenza. Young (3 months) and aged (18–20 months) adult mice were intranasally instilled with 12.5 PFU of influenza (strain: A/Puerto Rico/8/1934, PR8, H1N1) or (strain: A/Aichi/2/1968, HKx31, H3N2). (**A**,**B**) At select time points post infection BAL was collected from mice and (**A**) cell number or (**B**) protein concentration was quantified. (**C**) Lung tissue was collected at day 7 post infection. Wet weight (initial weight upon lung tissue removal) was quantified prior to incubation at 60 °C for 48 h to yield dry weight measurements. (**D**) Mice were instilled on day 7 with 3mg/mL of FITC-dextran and relative FITC fluorescence in plasma was assessed. Student’s *t*-test: * *p* < 0.05, ** *p* < 0.01, *** *p* < 0.001, and **** *p* < 0.0001. Similar results were obtained from at least three independent experiments, with N = 5 per group. Data are expressed as the mean ± SD.

Based on these histological findings, we next examined cell numbers present in bronchoalveolar lavage (BAL) fluid isolated from young and aged adult lung at select time points post H1N1 or H3N2 infection. In response to H1N1, there was a significant increase in cells present in BAL detected at day 3 post infection (Figure 2A). Cell number continued to increase in aged lung, with significantly heightened quantities detected at day 7 post infection (Figure 2A). We next evaluated cellular numbers present in young and aged lung in response to H3N2 infection. At day 3 post infection, there was a marked increase in cellular recruitment, with significantly higher numbers of cells quantified in young lung when compared to aged (Figure 2A). By day 7 post infection, cell numbers remained significantly elevated in aged H3N2 infected lung (Figure 2A). We next examined protein levels in young and aged adult BAL samples collected from mice at day 3 and 7 post H1N1 or H3N2 infection. At day 3 post infection there was a significant increase in protein detected in aged adult lung in response to influenza, with heightened levels detected in response to H3N2 (Figure 2B). Similarly, at day 7 post influenza, there was significantly increased protein concentrations present in aged lung, with notably higher levels quantified in H3N2 lung (Figure 2B). We next investigated the amount of lung water accumulation in young and aged adult lung in response to H1N1 or H3N2 infection. At day 7 post H1N1 or H3N2 infection there was a significant increase in water accumulation in aged adult lung, as illustrated by increased wet to dry lung ratio (Figure 2C). To examine potential changes in alveolar epithelial and endothelial permeability in young and aged murine lung in response to H1N1 or H3N2, we quantified relative fluorescence levels in plasma post lung instillation with FITC. In response to influenza, there was a marked increase in permeability, as illustrated by increased FITC fluorescence in aged influenza infected lung, with significantly higher levels detected in response to H3N2 (Figure 2D). 

#### 2.1.3. Dysregulated Type I IFN Signaling in Aged Lung

**Figure 3:**
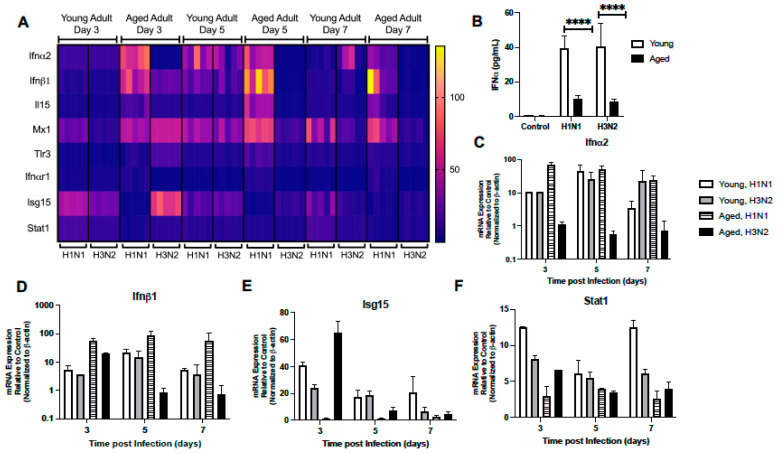
Dysregulated Type I IFN Signaling in Aged Lung. Young (3 months) and aged (18–20 months) adult mice were intranasally instilled with 12.5 PFU of influenza (strain: A/Puerto Rico/8/1934, PR8, H1N1) or (strain: A/Aichi/2/1968, HKx31, H3N2). (**A**) Lung tissue was collected from PBS treated or influenza infected young and aged mice at select time points post infection. Gene expression was assessed using the RT^2^ Profiler PCR Array, Mouse Antiviral Response, PAMM-122Z and results were quantified using analytical software provided by Qiagen Gene Globe. Please see Table 1 for gene abbreviations and Supplemental Tables S1 and S2 for complete details. (**B**) IFN-α expression in BAL was assessed by ELISA on day 3 post infection (student’s *t*-test: **** *p* < 0.0001). Representative gene expression of (**C**) Ifnα2 (two-way ANOVA, *p* < 0.0001), (**D**) Ifnβ1 (two-way ANOVA, *p* = 0.0361), (**E**) Isg15 (two-way ANOVA, *p* < 0.0001), and (**F**) Stat1 (two-way ANOVA, *p* < 0.0001). Similar results were obtained from at least three independent experiments, with N = 5 per group. Data are expressed as the mean ± SD.

Given the importance of antiviral signaling on host immune responses to influenza, we next examined the expression of several genes associated with the type I IFN signaling response in young and aged adult murine lung treated with PBS or influenza (H1N1 or H3N2) at days 3, 5, and 7 post infection. As shown in Figure 3A, there was a distinct pattern of genes elevated in both young and aged adult lung in response to each strain of influenza. On day 3, despite high Ifnα2 gene expression in aged H1N1 infected lung, we noted significantly diminished levels of IFNα present in BAL collected from H1N1 and H3N2 infected aged murine lung (Figure 3A–C). At day 3 post infection there was an increase in Ifnα2, Isg15, and Stat1 gene expression detected in young adult lung in response to either H1N1 or H3N2 (Figure 3A,C,E, and F; Supplemental Table S1). At day 5 of H1N1 or H3N2 infection, there was elevated expression of Ifnα2, Ifnβ1, Il-15, Mx1, and Tlr3 detected in young lung (Figure 3A,C,D, Supplemental Table S1). In response to H1N1 or H3N2, there was increased expression of Il-15, Tlr3, and Stat1 detected in young lung at day 7 of infection (Figure 3A,F, Supplemental Table S1). Examination of aged lung demonstrated differential expression patterns of genes associated with type I IFN signaling. Specifically, at day 3 post H1N1, there was increased Ifnα2 and Ifnβ1 and decreased Isg15 expression detected in aged lung (Figure 3A,C–E, Supplemental Table S2). In contrast, there was elevated expression of Isg15, Stat1, Mx1, and Tlr3 genes detected in aged lung at day 3 post H3N2 (Figure 3A,E,F, Supplemental Table S2). By days 5 and 7 of infection, there was a marked difference in gene expression with heightened expression of Mx1, Tlr3, Ifnα2, Ifnαr1, Ifnβ1, and Il-15 detected in aged lung in response to H1N1 (Figure 3A,C,D, Supplemental Table S2). In contrast, a similar elevation of Ifnα2, Ifnαr1, Ifnβ1, and Il-15 was not detected in aged lung in response to H3N2 (Figure 3A,C,D, Supplemental Table S2).

#### 2.1.4. Altered Expression of TLR Signaling Responsive Genes

**Figure 4:**
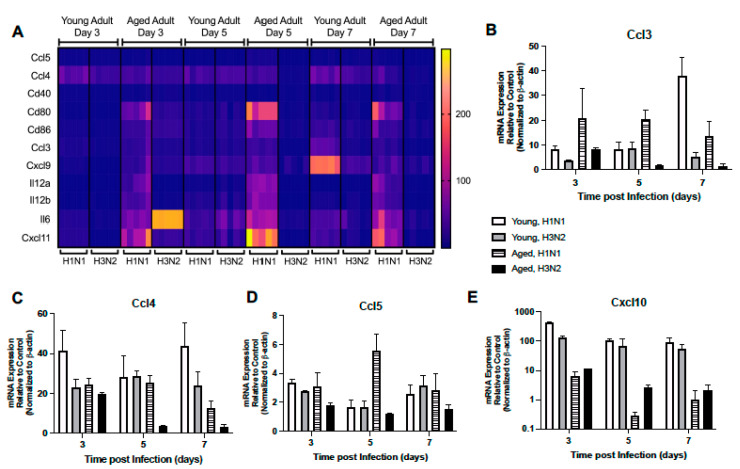
Altered Expression of TLR Signaling Responsive Genes. Young (3 months) and aged (18–20 months) adult mice were intranasally instilled with 12.5 PFU of influenza (strain: A/Puerto Rico/8/1934, PR8, H1N1) or (strain: A/Aichi/2/1968, HKx31, H3N2). Lung tissue was collected from PBS treated or influenza infected young and aged mice at select time points post infection. (**A**) Gene expression was assessed using the RT^2^ Profiler PCR Array, Mouse Antiviral Response, PAMM-122Z and results were quantified using analytical software provided by Qiagen Gene Globe. Please see Table 1 for gene abbreviations and Supplemental Tables S1 and S2 for complete list of results. Representative gene expression of (**B**) Ccl3 (two-way ANOVA, *p* < 0.0001), (**C**) Ccl4 (two-way ANOVA, *p* < 0.0001), (**D**) Ccl5 (two-way ANOVA, *p* < 0.0001), and (**E**) Cxcl10 (*p* < 0.0001). Similar results were obtained from at least three independent experiments, with N = 5 per group. Data are expressed as the mean ± SD.

We next investigated the expression pattern of TLR receptor signaling responsive genes in young and aged lung during H1N1 or H3N2 infection. In response to H1N1 or H3N2, there was a marked elevation of Ccl3, Ccl4, Ccl5, and Cxcl10 in young lung at day 3 post infection (Figure 4A–E, Supplemental Table S1). While some expression patterns remained elevated during infection, by day 5 there was increased Il-12a, Il-12b, Il-6, Cd80, and Cxcl11 expression that corresponded with elevated Il-15 levels in young adult H1N1 and H3N2 infected lung (Figure 4A, Supplemental Table S1). By day 7, expression of Ccl3, Cxcl9, Cd86, Il-6, Cd80, and Cxcl11 remained elevated in young adult lung in response to H1N1, while expression of Ccl5, Ccl4, and Cd40 remained elevated in response to either strain of influenza (Figure 4A–D, Supplemental Table S1). In aged adult lung, there was a significant upregulation of multiple TLR signaling responsive genes at day 3 post H1N1, with a marked increase in Cd80, Cxcl11, and Il-6 expression being detected (Figure 4A, Supplemental Table S1). Interestingly, by days 5 and 7, there was heightened expression of multiple genes, such as Cxcl9, Ccl3, Ccl4, Il-12a, Il-12b, Cd86, Cxcl11, and Cd80, detected in aged lung in response to H1N1 (Figure 4A–C, Supplemental Table S2). 

#### 2.1.5. Altered Expression of RIG-I-like Receptor Signaling

**Figure 5:**
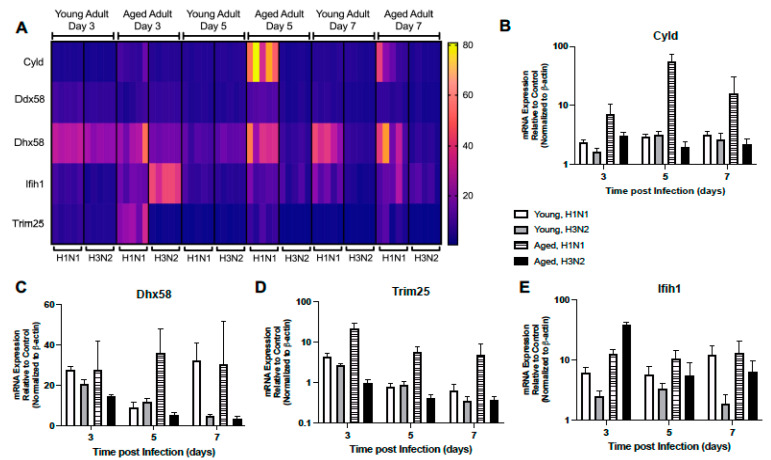
Altered Expression of RIG-I-Like Receptor Signaling. Young (3 months) and aged (18–20 months) adult mice were intranasally instilled with 12.5 PFU of influenza (strain: A/Puerto Rico/8/1934, PR8, H1N1) or (strain: A/Aichi/2/1968, HKx31, H3N2). Lung tissue was collected from PBS treated or influenza infected young and aged mice at select time points post infection. (**A**) Gene expression was assessed using the RT^2^ Profiler PCR Array, Mouse Antiviral Response, PAMM-122Z and results were quantified using analytical software provided by Qiagen Gene Globe. Please see Table 1 for gene abbreviations and Supplemental Tables S1 and S2 for complete list of results. Representative expression of (**B**) Cyld (two-way ANOVA, *p* < 0.0001), (**C**) Dhx58 (two-way ANOVA, *p* = 0.0058), (**D**) Trim25 (two-way ANOVA, *p* < 0.0001), and (**E**) Ifih1 (two-way ANOVA, *p* < 0.0001). Similar results were obtained from at least three independent experiments, with N = 5 per group. Data are expressed as the mean ± SD.

Given the importance of RIG-I signaling in host mediated immune response to influenza, we next examined the impact of age on lung responses to H1N1 or H3N2 infection. In young lung, there was similar expression patterns in H1N1 and H3N2 lung, with increased expression of Ddx58, Trim25, and Dhx58 detected on day 3 and Cyld expression on day 5 post infection (Figure 5A–D, Supplemental Table S1). When compared to H3N2, by day 7, Ddx58, Dhx58, and Ifih1 expression remained elevated in young H1N1 infected lung (Figure 5A,C,E, Supplemental Table S1). In contrast, there was altered expression in aged lung on day 3 post infection, with increased Dhx58, Cyld, Trim25, and Ddx58 detectable in aged H1N1 infected lung tissue (Figure 5A–D, Supplemental Table S2). Despite increased Ifih1 gene expression on day 3 post infection, diminished RIG-I like receptor signaling was observed in aged H3N2 infected lung on day 5 and 7 post infection (Figure 5A–E, Supplemental Table S2).

**Figure 6:**
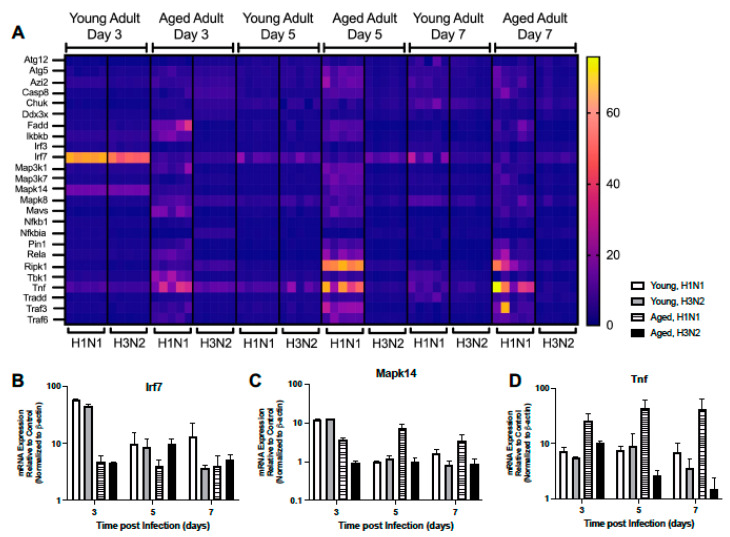
Altered Expression of RIG-I-Like Receptor Signaling. Young (3 months) and aged (18–20 months) adult mice were intranasally instilled with 12.5 PFU of influenza (strain: A/Puerto Rico/8/1934, PR8, H1N1) or (strain: A/Aichi/2/1968, HKx31, H3N2). Lung tissue was collected from PBS treated or influenza infected young and aged mice at select time points post infection. (**A**) Gene expression was assessed using the RT^2^ Profiler PCR Array, Mouse Antiviral Response, PAMM-122Z and results were quantified using analytical software provided by Qiagen Gene Globe. Please see Table 1 for gene abbreviations and Supplemental Tables S1 and S2 for complete list of results. Representative expression of (**B**) Irf7 (two-way ANOVA, *p* < 0.0001), (**C**) Mapk14 (two-way ANOVA, *p* < 0.0001), and (**D**) Tnf (two-way ANOVA, *p* = 0.00459). Similar results were obtained from at least three independent experiments, with N = 5 per group. Data are expressed as the mean ± SD.

We investigated the impact of aging on the expression of downstream RIG-I-like receptor signaling molecules. In young lung, there were comparable Pin1, Iκbκb, Mapk14, Map3k1, Rela, Irf7, Map3k7, and Nfκb1 expression patterns at days 3 and 5 post H1N1 or H3N2 infection (Figure 6A–C, Supplemental Table S1). In response to H1N1 or H3N2 infection, on day 5 post infection there were also similar expression patterns of Mavs, Traf3, and Traf6 detected in young lung (Figure 6A, Supplemental Table S1). Interestingly, by day 7 of infection, multiple genes were elevated in young H1N1 infected lung, such as Atg5, Mapk8, Tbk1, Casp8, and Ripk1, that were not similarly expressed in response to H3N2 (Figure 6A, Supplemental Table S1). In aged adult lung, there was a differential pattern of gene expression observed in response to H1N1 or H3N2 infection (Figure 6A). Specifically, at day 3, while the expression of Tbk1, Tnf, Mavs, and Traf6 were upregulated in response to H1N1, only the expression of Ddx3x, Chuk, Irf7, and Nfκbia were highly expressed in aged lung in response to H3N2 (Figure 6A,B,D, Supplemental Table S2). By days 5 and 7, heightened expression of Pin1, Traf6, Ripk1, Map3k7, Map3k1, Mapk14, Atg5, and TNF were observed in aged lung in response to H1N1 (Figure 6A,D, Supplemental Table S2). 

#### 2.1.6. Dysregulated Expression of TLR Receptors and Chaperones

**Figure 7:**
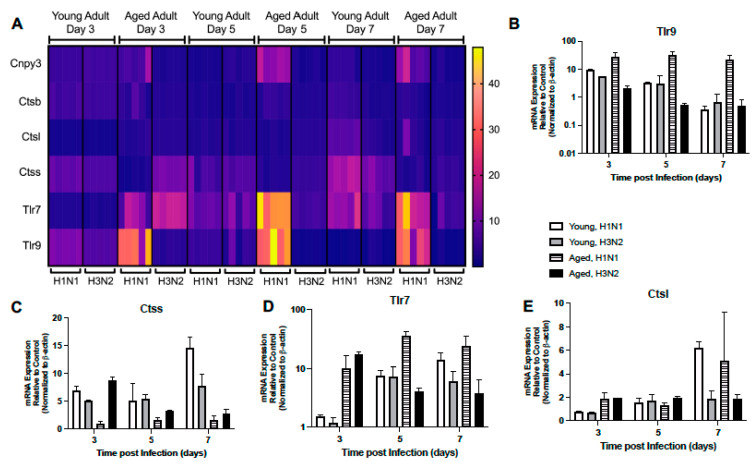
Dysregulated Expression of TLR Receptors and Chaperones. Young (3 months) and aged (18–20 months) adult mice were intranasally instilled with 12.5 PFU of influenza (strain: A/Puerto Rico/8/1934, PR8, H1N1) or (strain: A/Aichi/2/1968, HKx31, H3N2). Lung tissue was collected from PBS treated or influenza infected young and aged mice at select time points post infection. (**A**) Gene expression was assessed using the RT^2^ Profiler PCR Array, Mouse Antiviral Response, PAMM-122Z and results were quantified using analytical software provided by Qiagen Gene Globe. Please see Table 1 for gene abbreviations and Supplemental Tables S1 and S2 for complete list of results. Representative expression of (**B**) Tlr9 (two-way ANOVA, *p* = 0.0141), (**C**) Ctss (two-way ANOVA, *p* < 0.0001), (**D**) Tlr7 (two-way ANOVA, *p* = 0.00025), (**E**) Ctsl (two-way ANOVA, *p* = 0.0001). Similar results were obtained from at least three independent experiments, with N = 5 per group. Data are expressed as the mean ± SD.

We investigated if there was an age-associated alteration in the expression of TLR receptors and chaperones in lung during H1N1 or H3N2 infection. In young adult lung, there was increased expression of Tlr9, Cnpy3, and Ctsb at day 3 post H1N1 or H3N2 infection (Figure 7A,B, Supplemental Table S1). Similar expression patterns of Tlr7, Ctsl, and Ctss were observed in young lung at day 5 post H1N1 or H3N2 infection (Figure 7A, C–E, Supplemental Table S1). However, by day 7, elevated Ctsl, Ctsb, and Cnpy3 only remained elevated in young, H1N1 infected lung tissue (Figure 7A,C,E, Supplemental Table S1). In aged lung, Tlr9 expression was remained highly elevated during H1N1 infection (Figure 7A,B, Supplemental Table S2). Despite similar levels of Tlr7 on day 3 post H1N1 or H3N2 infection, gene expression levels remained heightened in aged lung on day 5 and 7 post H1N1 (Figure 7A,D, Supplemental Table S2). By days 5 and 7, heightened expression of Ctsl and Cnpy3 were detected in aged lung in response to H1N1 infection (Figure 7A,E, Supplemental Table S2). Taken together, these data demonstrate that the expression of TLR receptors, chaperones, and signaling molecules was dysregulated in aged lung in response to influenza. 

## 3. Discussion

The purpose of our current study was to examine the impact of chronological aging on antiviral signaling in murine lung in response to mouse-adapted strains of influenza, such as H1N1 or H3N2. The results of our research demonstrate distinct influenza strain specific histopathological changes occur in young and aged adult murine lung. There was increased cellular infiltration and inflammation present in H1N1 or H3N2 infected aged lung when compared to young. Similarly, there was heightened permeability and increased fluid accumulation in aged H1N1 or H3N2 infected lung tissue. An examination of the antiviral signaling pathways that might contribute to this phenotype illustrated that there was dysregulated type I IFN signaling as well as altered expression of TLR signaling receptors and responsive genes. Taken together, the data in our current study demonstrate that age and strain dependent antiviral signaling contribute to differential outcomes in the lung during influenza infection. 

Our results further demonstrate that decreased antiviral signaling in aged lung may contribute to increased permeability of aged lung and sustained weight loss during H3N2. Interferon production and expression of IFN stimulated genes play a critical role in the suppression of viral translocation and replication. Diminished antiviral signaling observed in aged lung in response to H3N2 infection can contribute to decreased transcriptional activation and impaired adaptive immune responses. By day 7 post H3N2 infection, there was a marked increase in lung injury and epithelial permeability in aged lung when compared to H1N1. As the expression of IFNs and their stimulated genes are critical for limiting viral replication, it is plausible that diminished expression of these genes in response to H3N2 may underlie this phenotype. 

In agreement with previous work, we observed dysregulated TLR expression in aged lung in response to H1N1 or H3N2 infection, which may further contribute to increased susceptibility of older persons to influenza [16,17,18,19,20,21,22,23,24,25,26]. Interestingly, when compared to young, while the expression of TLR receptors and signaling complexes were dysregulated in aged lung in response to H1N1, these expression patterns were further reduced in aged lung during H3N2 infection. Given the essential role for TLR signaling in mediating host antiviral responses, future work to examine the mechanisms that contribute to dysregulated TLR expression and downstream signaling will need to be performed.

It has been well accepted that mitochondria play a critical role in not only maintaining cellular energetics and homeostasis, but also are essential for modulating innate antiviral signaling [41,42,43]. Expression of MAVS on the mitochondrial surface can be significantly altered in response to changes in mitochondrial metabolism and mitochondrial damage [44,45]. Our findings expand upon previous work and demonstrate diminished expression of RIG-I receptor signaling as well as decreased downstream RIG-I signaling molecules, such as MAVS, was present in aged murine lung in response to H3N2 [27]. Interestingly, in response to H1N1, there was detectable upregulation of RIG-I receptor signaling and MAVS expression in aged lung by day 3, with expression remaining elevated at day 5. It is therefore plausible that different influenza subtypes, in addition to the impact of chronological aging, may contribute to the upregulation of specific genes within the RIG-I signaling cascades and differential gene expression patterns observed in aged lung in response to H1N1 or H3N2 infection. Of note, our study demonstrates that expression of RIG-I/MAVS regulatory molecules, such as TRIM25, were disproportionality increased in aged lung in response to H1N1 and not H3N2 infection. While our findings examine gene expression in response to influenza, the role of mitophagy induction, reactive oxygen species, and MAVS degradation will need to be examined in future studies to fully differentiate the impact of aging on this pathway and diminished MAVS expression in aged lung in response to H3N2 infection. Taken together, our results illustrate a potential role for dysregulated RIG-I signaling in decreased IFN production and antiviral signaling in aged lung in response to influenza.

Mitogen-activated protein kinases (MAPK) cascades play an important role in mediating extracellular responses to regulatory proteins and mediate inflammatory signaling in response to infectious stimuli. When compared to young, there was diminished upregulation of several MAPK in aged lung in response to influenza. MAPK, such as MAPK14/MAPK p38 play a critical role IFN induction and expression of IFN-stimulated genes, such as STAT1 [46]. Diminished expression in aged lung in response to H1N1 or H3N2 may further contribute to decreased IFN induction and production of additional antiviral cytokines. Additional work has demonstrated that inhibition of MAPK p38 can contribute to viral particle retention [47]. Specifically, in the absence of MAPK p38, there was increased translation of cellular and viral protein [48]. Given our findings, delayed MAPK p38 expression might also contribute to increased influenza titers and heightened injury to the aged lung in response to infection. 

There are several strengths and weaknesses of our current study. One strength of our study was that we used two distinct murine adapted strains of influenza to examine the impact of chronological aging on antiviral signaling. Another strength of our study was that chronologically aged animal models were utilized to investigate gene expression changes over a time course of infection. Similarly, our study, in addition to day 3, examined additional time points, such as days 5–7 of influenza infection that share similar clinical manifestations between mice and humans. One major limitation of our study was that we only present gene expression of antiviral immune signaling without protein levels or functional studies.

In summary, our current work provides a greater insight of antiviral signaling at days 3, 5, and 7 of H1N1 or H3N2 infection in young and aged adult murine lung. Using a murine model of mouse-adapted H1N1 and H3N2 infection, the results of our study demonstrate dysregulated and/or diminished activation of key signaling pathways in aged lung contribute to increased lung inflammation and morbidity and expand our current understanding of age-associated changes in antiviral signaling in the lung.

## 4. Materials and Methods

Mice: Young adult (3 months) and aged adult (18–20 months) male and female BALB/c mice were purchased from the NIA rodent facility (Charles River Laboratories). Upon receipt, mice were handled under identical husbandry conditions and fed certified commercial feed. Body weights were measured daily, and mice were humanely euthanized if they lost more than 15% of their starting body weight. The IACUC at Weill Cornell Medicine approved the use of animals in this study and methods were carried out in accordance with the relevant guidelines and regulations. No animals were used in the study if there was evidence of skin lesions, weight loss, or lymphadenopathy.

i. Influenza: Viral stocks: H1N1 (strain: A/Puerto Rico/8/1934, PR8, material #: 10100374, batch #: 4XP170531, EID_50_ per ml: 10^10.3^) and H3N2 (strain: A/Aichi/2/1968, HKx31, material #:10100375, batch #: 4XX171019, EID_50_ per ml: 10^10.5^) were purchased from Charles River (Norwich, CT).
ii. In Vivo Procedures and Tissue Collection: Influenza infection: All mice were anesthetized with isoflurane (5% for induction and 2% for maintenance) prior to intranasal instillation with 12.5 PFU of influenza (50 μL volume in PBS). Bronchoalveolar lavage (BAL): BAL was collected using previously published methods [49]. Briefly, 0.8-mL of PBS was slowly injected and aspirated 4 times prior to saving the recovered lavage fluid on ice. Lavage was clarified at 7000 rpm for 10 min at 4 °C. Viral titer assay of BAL: TCID_50_ in was calculated using the Viral ToxGlo Assay (Promega, Madison WI). Briefly, 3.16-fold serial dilutions of virus were plated for 24–48 h on >80% confluent MDCK cells. Upon visualization of cytopathic effect, ATP detection reagent was added, and luminescence was measured. Values were calculated by plotting net relative luminescence units (RLU) values after subtracting average blank wells against viral dilution. The TCID_50_ value is the reciprocal of the dilution that produced a 50% decline in ATP levels compared to untreated controls. Validated regression analysis was performed using GraphPad Prism. Protein quantification in BAL: Protein levels in clarified lavage were calculated using the BioRad protein assay (BioRad) per manufacturer’s instructions. IFNα ELISA: IFNα2 and 4 levels in clarified BAL were assessed by ELISA (ThermoFisher Scientific, Catalog # BMS6027) per manufacturer’s instructions. Lung tissue collection: At select time points of infection lung tissue was collected from control and influenza infected young and aged adult mice. Tissue was snap frozen or placed into Allprotect (Qiagen) for future analysis. FITC-Dextran Lung Permeability Assay: Young and aged adult mice were intranasally instilled with 50-μL of FITC-Dextran (3 mg/kg). After 1 h, blood was collected from euthanized mice, and plasma was isolated after centrifugation (7000 rpm, 10 min). Fluorescence was assessed (excitation 485, emission 528). Lung Wet to Dry Ratio: Lung tissue was collected from control and influenza infected young and aged adult mice. Lung tissue weight was assessed at harvest (wet weight) and after being placed in a 60 °C drying over for 48 h (dry weight). Histology: Mice were euthanized, and right lung tissue was collected for downstream analysis. To maintain architecture, left lung was distended with 1% low melting agarose and placed into cold formalin [50]. Tissue samples were processed, and H&E stained by the Translational Research Program at WCM Pathology and Laboratory of Medicine. Images were scanned using the EVOS FL Auto Imaging System (ThermoFisher Scientific). For all animal experiments, we used 5–10 mice per group and experiments were repeated at least three times.
iii. RNA Purification and Real Time PCR: RNA samples were extracted using the automated Maxwell RNA extraction protocol (Madison, WI). Samples were quantified and A_260/280_ ratios were recorded. Samples were reverse transcribed using the First Stand Synthesis Kit and quantified RT^2^ Profiler^TM^ Assays (RT^2^ Profiler PCR Array, Mouse Antiviral Response, PAMM-122Z). Results were quantified using analytical software provided by Qiagen Gene Globe.
iv. Statistical Analysis: Survival analysis between groups was calculated using the Mantel Cox test. Comparison of groups was performed using a two-tailed t-test and comparisons between groups were verified by one-way ANOVA. For two component comparisons (time post infection and age), two-way ANOVA was used to calculate statistical significance. All samples were independent and contained the same sample size for analysis. All data were analyzed using GraphPad Prism software (San Diego, CA). Statistical significance was considered by a * *p* < 0.05, **p < 0.01, *** *p* < 0.001, and **** *p* < 0.0001.

## Figures and Tables

**Table 1:** Symbol and gene names.

Symbol	Gene Name
Atg12	Autophagy-related 12
Atg5	Autophagy-related 5
Azi2	5-azacytidine induced gene 2
Casp8	Caspase 8
Ccl3	Chemokine (C-C motif) ligand 3
Ccl4	Chemokine (C-C motif) ligand 4
Ccl5	Chemokine (C-C motif) ligand 5
Cd40	CD40 antigen
Cd80	CD80 antigen
Cd86	CD86 antigen
Chuk	Conserved helix-loop-helix ubiquitous kinase
Cnpy3	Canopy 3 homolog
Ctsb	Cathepsin B
Ctsl	Cathepsin L
Ctss	Cathepsin S
Cxcl10	Chemokine (C-X-C motif) ligand 10
Cxcl9	Chemokine (C-X-C motif) ligand 9
Cyld	Cylindromatosis
Ddx3x	DEAD/H (Asp-Glu-Ala-Asp/His) box polypeptide 3
Ddx58	DEAD (Asp-Glu-Ala-Asp) box polypeptide 58
Dhx58	DEXH (Asp-Glu-X-His) box polypeptide 58
Fadd	Fas (TNFRSF6)-associated via death domain
Ifih1	Interferon induced with helicase C domain 1
Ifnα2	Interferon alpha 2
Ifnαr1	Interferon (alpha and beta) receptor 1
Ifnβ1	Interferon beta 1
Ikbkb	Inhibitor of kappaB kinase beta
Il12a	Interleukin 12A
Il12b	Interleukin 12b
Il15	Interleukin 15
Il6	Interleukin 6
Irf3	Interferon regulatory factor 3
Irf7	Interferon regulatory factor 7
Isg15	ISG15 ubiquitin-like modifier
Map3k1	Mitogen-activated protein 3 kinase 1
Map3k7	Mitogen-activated protein 3 kinase 7
Mapk14	Mitogen-activated protein kinase 14
Mapk8	Mitogen-activated protein kinase 8
Mavs	Mitochondrial antiviral signaling protein
Mx1	Myxovirus (Influenza virus) resistance 1
Nfκb1	Nuclear factor of kappa light polypeptide gene enhancer in B- cells 1, p105
Nfκbia	Nuclear factor of kappa light polypeptide gene enhancer in B- cells inhibitor, alpha
Pin1	Protein (peptidyl-proyl cis/trans isomerase) NIMA-interacting 1
Rela	V-rel reticuloendotheliosis viral oncogene homolog A
Ripk1	Receptor (TNFRSF)-interacting serine-threonine kinase 1
Stat1	Signal transducer and activator of transcription 1
Tbk1	TANK-binding kinase 1
Tlr3	Toll-like receptor 3
Tlr9	Toll-like receptor 9
Tnf	Tumor necrosis factor
Tradd	TNFRSF1A-associated via death domain
Traf3	Tnf receptor-associated factor 3
Traf6	Tnf receptor-associated factor 6
Trim25	Tripartite motif-containing 25

## Data Availability

Data presented in this study is provided within the article, with complete gene expression values listed in Supplemental Tables S1 and S2.

## Supplementary Materials

The following are available online at https://www.mdpi.com/article/10.3390/ijms222212097/s1.
