# Supplementary material for: Antiviral Gene Expression in Young and Aged Murine Lung during H1N1 and H3N2
Source: Int J Mol Sci. 2021 Nov 9;22(22):12097. doi: 10.3390/ijms222212097 (PMC8618707; doi:10.3390/ijms222212097)
Supplement: Supplementary file 1 [file ijms-22-12097-s001.zip › ijms-1418435-supplementary.pdf]

Supplemental Table S1		Fold Change (comparing to control group)			
**Young**		**H1N1, Day 3**		**H3N2, Day 3**	
Refseq	Symbol	Fold Change	P value	Fold Change	P value
NM_026217	Atg12	0.58	0.000035	0.45	0.000000
NM_053069	Atg5	1.31	0.908478	1.89	0.009057
NM_013727	Azi2	3.81	0.000000	2.53	0.000025
NM_009812	Casp8	1.29	0.001144	0.95	0.000930
NM_011337	Ccl3	8.06	0.000291	3.63	0.000002
NM_013652	Ccl4	40.52	0.000491	22.71	0.000065
NM_013653	Ccl5	3.36	0.000009	2.79	0.000000
NM_011611	Cd40	3.08	0.000056	2.34	0.000330
NM_009855	Cd80	2.74	0.005140	1.73	0.012698
NM_019388	Cd86	5.19	0.000001	2.73	0.000000
NM_007700	Chuk	0.74	0.000121	0.64	0.000008
NM_028065	Cnpy3	3.44	0.000000	3.98	0.000007
NM_007798	Ctsb	4.25	0.000324	3.72	0.000000
NM_009984	Ctsl	0.75	0.000000	0.68	0.000000
NM_021281	Ctss	6.95	0.000014	5.12	0.000000
NM_021274	Cxcl10	440.01	0.000001	135.37	0.000012
NM_008599	Cxcl9	4.39	0.001385	1.23	0.000000
NM_173369	Cyld	2.39	0.000203	1.64	0.388438
NM_010028	Ddx3x	1.75	0.000044	1.57	0.000011
NM_172689	Ddx58	6.77	0.000040	4.69	0.000004
NM_030150	Dhx58	27.79	0.000000	20.60	0.000002
NM_010175	Fadd	1.98	0.000000	2.19	0.018313
NM_027835	Ifih1	6.11	0.000856	2.43	0.042284
NM_010503	Ifnα2	10.56	0.000000	10.42	0.000000
NM_010508	Ifnar1	2.28	0.001267	2.51	0.000002
NM_010510	Ifnβ1	5.11	0.020590	3.58	0.000000
NM_010546	Iκbκb	3.73	0.000000	4.08	0.000003
NM_008351	Il12a	0.94	0.000022	1.49	0.956070
NM_001303244	Il12b	2.03	0.119093	1.48	0.858082
NM_008357	Il15	3.00	0.000015	2.00	0.001088
NM_001314054	Il6	9.98	0.000000	3.40	0.003600
NM_016849	Irf3	2.08	0.000227	2.55	0.028848
NM_016850	Irf7	59.41	0.000000	44.70	0.000001
NM_015783	Isg15	40.86	0.000000	23.96	0.000001
NM_011945	Map3k1	2.43	0.002034	2.73	0.000003

NM_172688	Map3k7	1.74	0.011299	1.78	0.000041
NM_011951	Mapk14	12.57	0.000000	12.69	0.000030
NM_016700	Mapk8	1.72	0.088715	1.82	0.000000
NM_144888	Mavs	10.70	0.000022	10.56	0.000000
NM_010846	Mx1	22.00	0.000028	6.41	0.000738
NM_008689	Nfκb1	1.68	0.003684	1.38	0.042398
NM_010907	Nfκbia	1.62	0.513729	0.81	0.000000
NM_023371	Pin1	1.41	0.021038	1.44	0.495693
NM_009045	Rela	2.05	0.000000	1.96	0.002635
NM_009068	Ripk1	1.26	0.000223	0.77	0.000046
NM_009283	Stat1	12.48	0.000000	8.16	0.000000
NM_019786	Tbk1	2.58	0.001809	1.68	0.000081
NM_126166	Tlr3	1.91	0.003728	0.88	0.036518
NM_133211	Tlr7	1.52	0.775943	1.18	0.076933
NM_031178	Tlr9	9.54	0.000001	5.42	0.000000
NM_013693	Tnf	7.24	0.000110	5.52	0.000000
NM_001033161	Tradd	1.07	0.006207	1.27	0.085779
NM_011632	Traf3	1.38	0.210129	1.18	0.135206
NM_009424	Traf6	0.77	0.000054	0.61	0.000012
NM_009546	Trim25	4.37	0.002060	2.70	0.000013

		**Fold Change (comparing to control group)**			
**Young**		**H1N1, Day 5**		**H3N2, Day 5**	
Refseq	Symbol	Fold Change	P value	Fold Change	P value
NM_026217	Atg12	1.81	0.009167	2.22	0.009167
NM_053069	Atg5	2.47	0.001583	2.69	0.001583
NM_013727	Azi2	2.30	0.002449	2.62	0.002449
NM_009812	Casp8	2.11	0.002089	1.80	0.002089
NM_011337	Ccl3	7.89	0.010489	8.53	0.010489
NM_013652	Ccl4	26.28	0.013596	28.65	0.013596
NM_013653	Ccl5	1.59	0.035940	1.61	0.035940
NM_011611	Cd40	2.70	0.009104	3.00	0.009104
NM_009855	Cd80	8.71	0.007068	6.48	0.007068
NM_019388	Cd86	8.01	0.014250	10.92	0.014250
NM_007700	Chuk	3.54	0.003820	0.21	0.003820
NM_028065	Cnpy3	0.97	0.009970	1.78	0.009970
NM_007798	Ctsb	1.25	0.014200	1.24	0.014200
NM_009984	Ctsl	1.53	0.027086	1.68	0.027086
NM_021281	Ctss	4.52	0.045000	5.49	0.045000

NM_021274	Cxcl10	105.14	0.002736	57.30	0.002736
NM_008599	Cxcl9	24.68	0.001930	18.85	0.001930
NM_173369	Cyld	2.92	0.001564	3.20	0.001564
NM_010028	Ddx3x	1.25	0.002594	1.49	0.002594
NM_172689	Ddx58	2.98	0.002494	2.89	0.002494
NM_030150	Dhx58	8.69	0.007154	11.79	0.007154
NM_010175	Fadd	1.72	0.016944	1.43	0.016944
NM_027835	Ifih1	5.59	0.004189	3.36	0.004189
NM_010503	Ifna2	12.76	0.024895	9.60	0.024895
NM_010508	Ifnar1	0.93	0.032493	0.87	0.032493
NM_010510	Ifnb1	21.12	0.008928	12.41	0.008928
NM_010546	Ikbkb	1.50	0.006896	1.48	0.006896
NM_008351	Il12a	3.40	0.002044	3.23	0.002044
NM_001303244	Il12b	9.16	0.003214	6.52	0.003214
NM_008357	Il15	4.66	0.002184	4.59	0.002184
NM_001314054	Il6	22.27	0.002130	34.72	0.002130
NM_016849	Irf3	1.30	0.014218	1.37	0.014218
NM_016850	Irf7	8.43	0.041062	8.25	0.041062
NM_015783	Isg15	16.30	0.010774	18.53	0.010774
NM_011945	Map3k1	1.13	0.000957	1.28	0.000957
NM_172688	Map3k7	1.29	0.001321	1.45	0.001321
NM_011951	Mapk14	10.99	0.002277	11.21	0.002277
NM_016700	Mapk8	3.39	0.000850	4.43	0.000850
NM_144888	Mavs	1.25	0.016450	1.05	0.016450
NM_010846	Mx1	37.24	0.002373	28.65	0.002373
NM_008689	Nfkb1	0.80	0.006126	0.79	0.006126
NM_010907	Nfkbia	0.91	0.004525	0.68	0.004525
NM_023371	Pin1	0.71	0.038598	0.73	0.038598
NM_009045	Rela	0.38	0.396582	0.49	0.396582
NM_009068	Ripk1	1.59	0.001822	1.26	0.001822
NM_009283	Stat1	5.87	0.002779	5.37	0.002779
NM_019786	Tbk1	1.97	0.003597	2.31	0.003597
NM_126166	Tlr3	4.55	0.000414	4.13	0.000414
NM_133211	Tlr7	7.35	0.001182	6.74	0.001182
NM_031178	Tlr9	3.30	0.000903	2.24	0.000903
NM_013693	Tnf	7.39	0.002046	7.07	0.002046
NM_001033161	Tradd	1.46	0.002049	1.72	0.002049
NM_011632	Traf3	2.20	0.006168	1.75	0.006168
NM_009424	Traf6	1.44	0.003683	1.24	0.003683

NM_009546	Trim25	0.75	0.073871	0.84	0.073871

		Fold Change (comparing to control group)			
**Young**		**H1N1, Day 7**		**H3N2, Day 7**	
Refseq	Symbol	Fold Change	P value	Fold Change	P value
NM_026217	Atg12	1.01	0.009167	2.29	0.009167
NM_053069	Atg5	4.08	0.001583	1.81	0.001583
NM_013727	Azi2	5.01	0.002449	1.77	0.002449
NM_009812	Casp8	3.77	0.002089	1.69	0.002089
NM_011337	Ccl3	37.03	0.010489	4.90	0.010489
NM_013652	Ccl4	42.60	0.013596	23.26	0.013596
NM_013653	Ccl5	2.49	0.035940	3.12	0.035940
NM_011611	Cd40	2.65	0.009104	2.68	0.009104
NM_009855	Cd80	8.81	0.007068	2.75	0.007068
NM_019388	Cd86	18.54	0.014250	7.47	0.014250
NM_007700	Chuk	7.22	0.003820	4.36	0.003820
NM_028065	Cnpy3	2.41	0.009970	0.83	0.009970
NM_007798	Ctsb	3.47	0.014200	1.34	0.014200
NM_009984	Ctsl	6.21	0.027086	1.76	0.027086
NM_021281	Ctss	14.53	0.045000	7.58	0.045000
NM_021274	Cxcl10	85.58	0.002736	53.16	0.002736
NM_008599	Cxcl9	183.71	0.001930	24.99	0.001930
NM_173369	Cyld	3.15	0.001564	2.63	0.001564
NM_010028	Ddx3x	1.97	0.002594	1.22	0.002594
NM_172689	Ddx58	3.81	0.002494	1.87	0.002494
NM_030150	Dhx58	31.38	0.007154	4.48	0.007154
NM_010175	Fadd	0.69	0.016944	0.55	0.016944
NM_027835	Ifih1	11.27	0.004189	1.70	0.004189
NM_010503	Ifna2	1.35	0.024895	3.66	0.024895
NM_010508	Ifnar1	0.94	0.032493	0.45	0.032493
NM_010510	Ifnb1	5.08	0.008928	2.04	0.008928
NM_010546	Ikbkb	1.49	0.006896	0.60	0.006896
NM_008351	Il12a	0.77	0.002044	1.30	0.002044
NM_001303244	Il12b	4.47	0.003214	2.87	0.003214
NM_008357	Il15	4.48	0.002184	2.60	0.002184
NM_001314054	Il6	29.72	0.002130	9.11	0.002130
NM_016849	Irf3	2.47	0.014218	1.58	0.014218
NM_016850	Irf7	9.65	0.041062	3.70	0.041062
NM_015783	Isg15	17.83	0.010774	5.84	0.010774

NM_011945	Map3k1	1.08	0.000957	0.64	0.000957
NM_172688	Map3k7	2.16	0.001321	1.53	0.001321
NM_011951	Mapk14	1.58	0.002277	0.83	0.002277
NM_016700	Mapk8	7.18	0.000850	2.89	0.000850
NM_144888	Mavs	0.32	0.016450	0.51	0.016450
NM_010846	Mx1	36.71	0.002373	9.47	0.002373
NM_008689	Nfkb1	0.89	0.006126	0.79	0.006126
NM_010907	Nfkbia	1.06	0.004525	1.10	0.004525
NM_023371	Pin1	0.64	0.038598	0.34	0.038598
NM_009045	Rela	0.16	0.396582	0.19	0.396582
NM_009068	Ripk1	2.48	0.001822	0.90	0.001822
NM_009283	Stat1	12.49	0.002779	6.07	0.002779
NM_019786	Tbk1	6.05	0.003597	1.63	0.003597
NM_126166	Tlr3	5.07	0.000414	2.75	0.000414
NM_133211	Tlr7	13.25	0.001182	5.42	0.001182
NM_031178	Tlr9	0.35	0.000903	0.46	0.000903
NM_013693	Tnf	6.26	0.002046	3.30	0.002046
NM_001033161	Tradd	5.13	0.002049	1.83	0.002049
NM_011632	Traf3	0.90	0.006168	1.03	0.006168
NM_009424	Traf6	0.72	0.003683	0.69	0.003683
NM_009546	Trim25	0.58	0.073871	0.33	0.073871

Supplemental Table S2		Fold Change (comparing to control group)			
**Aged**		**H1N1, Day 3**		**H3N2, Day 3**	
Refseq	Symbol	Fold Change	P value	Fold Change	P value
NM_026217	Atg12	1.07	0.167224	1.77	0.000001
NM_053069	Atg5	5.04	0.076311	1.28	0.000003
NM_013727	Azi2	5.67	0.084464	1.59	0.000001
NM_009812	Casp8	4.94	0.094489	2.15	0.000000
NM_011337	Ccl3	12.43	0.048352	8.42	0.000002
NM_013652	Ccl4	27.56	0.002915	19.58	0.000000
NM_013653	Ccl5	2.94	0.010237	1.82	0.000008
NM_011611	Cd40	3.46	0.009657	3.83	0.000008
NM_009855	Cd80	55.80	0.049687	14.34	0.000027
NM_019388	Cd86	22.36	0.016512	18.58	0.000001
NM_007700	Chuk	2.30	0.000736	4.14	0.000000
NM_028065	Cnpy3	5.45	0.078629	1.37	0.000335
NM_007798	Ctsb	2.61	0.079183	1.72	0.000002
NM_009984	Ctsl	1.78	0.006152	1.96	0.000000
NM_021281	Ctss	0.86	0.032015	8.77	0.000000
NM_021274	Cxcl10	6.20	0.013806	11.37	0.000000
NM_008599	Cxcl9	21.02	0.111288	11.73	0.000009
NM_173369	Cyld	6.68	0.024942	3.12	0.000059
NM_010028	Ddx3x	1.82	0.004326	3.19	0.000000
NM_172689	Ddx58	3.89	0.022146	5.36	0.000001
NM_030150	Dhx58	25.24	0.032887	14.53	0.000002
NM_010175	Fadd	15.93	0.046163	0.76	0.011177
NM_027835	Ifih1	12.40	0.001418	38.75	0.000011
NM_010503	Ifna2	66.87	0.000989	1.09	0.002186
NM_010508	Ifnar1	3.61	0.015700	0.47	0.994751
NM_010510	Ifnb1	52.21	0.000667	19.26	0.000007
NM_010546	Ikbkb	12.64	0.010021	0.95	0.001703
NM_008351	Il12a	34.21	0.042577	1.54	0.001664
NM_001303244	Il12b	31.35	0.008880	2.96	0.000212
NM_008357	Il15	13.72	0.024378	5.84	0.000006
NM_001314054	Il6	53.33	0.000438	241.00	0.000000
NM_016849	Irf3	0.91	0.037109	1.23	0.000012
NM_016850	Irf7	4.71	0.001056	4.25	0.000000
NM_015783	Isg15	1.11	0.000259	64.65	0.000002

NM_011945	Map3k1	7.18	0.080581	0.96	0.005113
NM_172688	Map3k7	1.40	0.050390	1.89	0.000043
NM_011951	Mapk14	5.59	0.041642	0.92	0.001067
NM_016700	Mapk8	9.18	0.101813	4.73	0.000004
NM_144888	Mavs	13.68	0.005964	0.55	0.320724
NM_010846	Mx1	39.22	0.000018	50.23	0.000000
NM_008689	Nfkb1	1.59	0.009520	0.92	0.000303
NM_010907	Nfkbia	0.40	0.010442	3.78	0.000000
NM_023371	Pin1	3.45	0.044812	0.71	0.020087
NM_009045	Rela	8.13	0.002650	0.62	0.071392
NM_009068	Ripk1	5.89	0.060569	5.68	0.000047
NM_009283	Stat1	2.78	0.023537	6.56	0.000000
NM_019786	Tbk1	14.80	0.006980	1.17	0.000089
NM_126166	Tlr3	3.15	0.048977	14.16	0.000000
NM_031178	Tlr9	26.04	0.008681	2.10	0.000495
NM_013693	Tnf	23.70	0.007399	10.05	0.000035
NM_001033161	Tradd	1.68	0.014624	1.38	0.000075
NM_011632	Traf3	4.73	0.032041	1.22	0.000989
NM_009424	Traf6	4.75	0.005715	0.92	0.002904
NM_009546	Trim25	20.74	0.009167	0.96	0.013456

		**Fold Change (comparing to control group)**			
**Aged**		**H1N1, Day 5**		**H3N2, Day 5**	
Refseq	Symbol	Fold Change	P value	Fold Change	P value
NM_026217	Atg12	2.21	0.000363	1.24	0.089758
NM_053069	Atg5	9.73	0.000319	1.36	0.10439
NM_013727	Azi2	11.37	0.000746	1.67	0.076372
NM_009812	Casp8	7.95	0.000105	2.10	0.01459
NM_011337	Ccl3	20.42	0.000084	2.07	0.000326
NM_013652	Ccl4	25.28	0.000077	3.43	0.00099
NM_013653	Ccl5	5.48	0.000002	1.20	0.001276
NM_011611	Cd40	1.91	0.003548	2.39	0.007736
NM_009855	Cd80	150.06	0.000024	2.91	0.01817
NM_019388	Cd86	45.86	0.000155	4.11	0.024384
NM_007700	Chuk	2.84	0.007888	2.61	0.022921
NM_028065	Cnpy3	12.76	0.00109	0.98	0.239538
NM_007798	Ctsb	2.51	0.0085	1.46	0.007883
NM_009984	Ctsl	1.34	0.001194	1.98	0.001847

NM_021281	Ctss	1.57	0.000209	3.21	0.000643
NM_021274	Cxcl10	0.28	0.040541	2.53	0.000002
NM_008599	Cxcl9	29.53	0.002001	6.14	0.051034
NM_173369	Cyld	53.30	0.003659	1.97	0.022535
NM_010028	Ddx3x	1.95	0.000747	1.59	0.063401
NM_172689	Ddx58	9.32	0.000422	2.32	0.036595
NM_030150	Dhx58	34.65	0.000336	5.01	0.010556
NM_010175	Fadd	8.11	0.001568	0.39	0.155297
NM_027835	Ifih1	10.11	0.000109	4.69	0.087039
NM_010503	Ifna2	47.78	0.000056	0.54	0.881631
NM_010508	Ifnar1	4.67	0.000807	0.54	0.950679
NM_010510	Ifnb1	85.83	0.004843	0.79	0.362657
NM_010546	Ikbkb	7.52	0.000172	0.81	0.343885
NM_008351	Il12a	64.92	0.000573	1.09	0.082908
NM_001303244	Il12b	59.09	0.001506	0.54	0.347476
NM_008357	Il15	38.92	0.000048	1.26	0.109517
NM_001314054	Il6	90.34	0.000001	5.93	0.017287
NM_016849	Irf3	1.34	0.010448	1.10	0.18239
NM_016850	Irf7	3.93	0.002714	9.46	0.00958
NM_015783	Isg15	1.26	0.000174	7.14	0.019733
NM_011945	Map3k1	11.29	0.000011	0.68	0.529506
NM_172688	Map3k7	10.50	0.000345	1.40	0.068773
NM_011951	Mapk14	7.33	0.004009	0.93	0.194268
NM_016700	Mapk8	6.46	0.00056	2.33	0.061977
NM_144888	Mavs	7.89	0.003556	0.55	0.987207
NM_010846	Mx1	65.93	0.000025	10.44	0.010878
NM_008689	Nfkb1	2.96	0.00002	0.74	0.350668
NM_010907	Nfkbia	0.60	0.000057	1.44	0.000225
NM_023371	Pin1	6.45	0.000517	0.49	0.651667
NM_009045	Rela	13.22	0.0024	0.47	0.697192
NM_009068	Ripk1	54.69	0.000338	2.87	0.00474
NM_009283	Stat1	3.97	0.000043	3.39	0.001902
NM_019786	Tbk1	4.25	0.007664	0.63	0.661547
NM_126166	Tlr3	13.34	0.000883	3.05	0.022304
NM_031178	Tlr9	32.75	0.005728	0.53	0.658943
NM_013693	Tnf	41.44	0.004943	2.59	0.014997
NM_001033161	Tradd	4.40	0.000507	1.46	0.029655
NM_011632	Traf3	19.12	0.000026	0.70	0.374617
NM_009424	Traf6	9.42	0.011754	1.21	0.040031

NM_009546	Trim25	5.24	0.016997	0.41	0.154941

		Fold Change (comparing to control group)			
**Aged**		**H1N1, Day 7**		**H3N2, Day 7**	
Refseq	Symbol	Fold Change	P value	Fold Change	P value
NM_026217	Atg12	1.37	0.004059	1.93	0.216154
NM_053069	Atg5	5.04	0.028275	1.28	0.349296
NM_013727	Azi2	5.67	0.009841	1.59	0.321506
NM_009812	Casp8	4.94	0.10244	2.15	0.212475
NM_011337	Ccl3	12.43	0.010451	1.51	0.267389
NM_013652	Ccl4	17.36	0.05735	2.90	0.114053
NM_013653	Ccl5	2.61	0.011294	1.50	0.029204
NM_011611	Cd40	3.52	0.006486	2.27	0.104053
NM_009855	Cd80	68.79	0.036204	2.29	0.317442
NM_019388	Cd86	22.47	0.042154	4.75	0.187827
NM_007700	Chuk	2.72	0.000472	3.62	0.156716
NM_028065	Cnpy3	8.30	0.052245	0.85	0.466441
NM_007798	Ctsb	1.57	0.046721	1.15	0.196664
NM_009984	Ctsl	2.18	0.194591	1.82	0.139638
NM_021281	Ctss	1.41	0.023059	2.64	0.133333
NM_021274	Cxcl10	0.64	0.126034	1.97	0.072693
NM_008599	Cxcl9	23.37	0.003573	12.13	0.104207
NM_173369	Cyld	12.13	0.077686	2.19	0.145846
NM_010028	Ddx3x	1.46	0.006762	1.33	0.304651
NM_172689	Ddx58	3.91	0.006309	1.57	0.240631
NM_030150	Dhx58	24.10	0.063364	3.20	0.151315
NM_010175	Fadd	5.14	0.018433	0.39	0.955542
NM_027835	Ifih1	11.51	0.019081	5.47	0.161447
NM_010503	Ifna2	21.80	0.008824	0.53	0.576995
NM_010508	Ifnar1	4.00	0.044693	0.38	0.619121
NM_010510	Ifnb1	39.04	0.097081	0.51	0.621738
NM_010546	Ikbkb	4.36	0.000412	0.62	0.600489
NM_008351	Il12a	34.03	0.033564	1.23	0.289163
NM_001303244	Il12b	28.37	0.016491	0.57	0.611809
NM_008357	Il15	11.30	0.073924	1.22	0.340881
NM_001314054	Il6	70.74	0.032437	5.75	0.250601
NM_016849	Irf3	1.04	0.055585	1.61	0.113259
NM_016850	Irf7	3.54	0.033846	5.18	0.050924

NM_015783	Isg15	2.11	0.041097	4.17	0.163555
NM_011945	Map3k1	3.35	0.027217	0.71	0.501744
NM_172688	Map3k7	2.33	0.115906	1.19	0.272624
NM_011951	Mapk14	3.28	0.012924	0.81	0.429777
NM_016700	Mapk8	5.53	0.001273	2.25	0.302945
NM_144888	Mavs	4.20	0.006813	0.46	0.92205
NM_010846	Mx1	45.56	0.015522	5.22	0.135181
NM_008689	Nfkb1	1.64	0.005063	0.76	0.42315
NM_010907	Nfkbia	0.40	0.000616	1.63	0.002218
NM_023371	Pin1	4.00	0.061728	0.36	0.634748
NM_009045	Rela	6.71	0.135584	0.31	0.43923
NM_009068	Ripk1	17.98	0.059513	2.47	0.152838
NM_009283	Stat1	2.49	0.006149	3.84	0.103277
NM_019786	Tbk1	0.92	0.195991	0.63	0.584796
NM_126166	Tlr3	6.57	0.005666	2.59	0.195362
NM_031178	Tlr9	21.16	0.004944	0.44	0.81537
NM_013693	Tnf	36.13	0.018263	1.35	0.323769
NM_001033161	Tradd	3.81	0.039556	1.85	0.129599
NM_011632	Traf3	9.84	0.299483	0.75	0.434129
NM_009424	Traf6	6.90	0.014614	0.89	0.330538
NM_009546	Trim25	3.62	0.105305	0.37	0.482317
